# Intrauterine Insemination (IUI) Outcomes With Total Progressive Motile Sperm Count (TPMSC) Above and Below 5 Million

**DOI:** 10.7759/cureus.82580

**Published:** 2025-04-19

**Authors:** Cem Inceoglu, Enis Ozkaya, Muserref Banu Yilmaz

**Affiliations:** 1 Department of Obstetrics and Gynecology, Sirnak State Hospital, Sirnak, TUR; 2 Department of Obstetrics and Gynecology, Kirklareli University, Kirklareli, TUR; 3 Department of Obstetrics and Gynecology, University of Health Sciences Türkiye, Zeynep Kamil Women’s and Children’s Diseases Training and Research Hospital, İstanbul, TUR

**Keywords:** intrauterine insemination (iui), oligoasthenospermia, pregnancy outcomes, total progressive motile sperm count (tpmsc), unexplained female infertility

## Abstract

Objective: The study aims to investigate the impact of total progressive motile sperm count (TPMSC) on pregnancy outcomes in couples diagnosed with unexplained infertility undergoing intrauterine insemination (IUI).

Materials and methods: A retrospective analysis was conducted on 122 IUI cycles from 98 couples with unexplained infertility who received treatment at Zeynep Kamil Women’s and Children’s Diseases Training and Research Hospital between 2021 and 2022. The couples were divided into two groups based on TPMSC: below 5 million and 5 million or above. Key pregnancy outcomes, including chemical pregnancy, clinical pregnancy, and live birth rates, were evaluated and compared between the groups.

Results: Of the 122 IUI cycles analyzed, 69 had a TPMSC of 5 million or above, and 53 cycles had a TPMSC below 5 million. The chemical pregnancy, clinical pregnancy, and live birth rates were significantly higher in the group with TPMSC above 5 million (34.8%, 31.9%, and 23.2%, respectively) compared to the TPMSC below 5 million group, which had no pregnancies. The findings demonstrate that TPMSC is a critical predictor of IUI success, with a TPMSC threshold of 5 million associated with significantly higher pregnancy outcomes.

Conclusion: This study highlights that a TPMSC below 5 million is associated with poor IUI outcomes, suggesting that IUI may not be a cost-effective treatment for couples with severe oligoasthenospermia. For these couples, alternative treatments like in vitro fertilization (IVF) may be more appropriate. The findings provide valuable insights for improving the management and treatment strategies for unexplained infertility.

## Introduction

Unexplained infertility is a condition in which no clear cause for a couple's inability to conceive can be identified despite thorough diagnostic testing. It accounts for a significant portion of infertility cases, affecting approximately 10-15% of couples worldwide [1]. While many factors contribute to infertility, unexplained infertility remains a challenging diagnosis as no specific etiology can be pinpointed, leaving physicians with limited options for targeted treatments [2]. The management of unexplained infertility often involves assisted reproductive technologies (ART), such as intrauterine insemination (IUI) and in vitro fertilization (IVF), which aim to improve the chances of conception [3].

Intrauterine insemination is a less invasive and less costly option than IVF and is often the first line of treatment for unexplained infertility [4]. The success of IUI depends on various factors, including the age of the woman, the quality of sperm, the timing of ovulation, and the overall health of both partners [5]. However, one of the key factors that can influence IUI outcomes is the male partner’s sperm quality, particularly the total progressive motile sperm count (TPMSC). TPMSC is considered a crucial parameter in determining the potential success of IUI, as it directly correlates with the sperm’s ability to reach and fertilize the egg [4].

The role of TPMSC in predicting the success of IUI remains an area of ongoing research. Previous studies have suggested that a higher TPMSC may increase the likelihood of pregnancy, whereas a lower count may decrease the chances of successful conception [5]. However, there is still no consensus on the optimal TPMSC threshold that would predict successful outcomes in IUI cycles, especially in patients with unexplained infertility [2].

The TPMSC cutoff for successful IUI has been reported, but the suggested thresholds have varied significantly and need further refinement [6]. Clearly defining this threshold would improve patient selection by helping to identify couples who may not be likely to conceive through IUI. This would allow them to transition to more advanced ART options, minimizing the physical, emotional, and financial challenges associated with the IUI process.

This study aims to explore the impact of TPMSC on pregnancy outcomes in couples diagnosed with unexplained infertility undergoing IUI treatment. By evaluating the chemical pregnancy, clinical pregnancy, and live birth rates of TPMSC levels, this research seeks to provide valuable insights into the role of sperm count in predicting IUI success and inform clinical decision-making in the management of unexplained infertility.

## Materials and methods

This retrospective study included 98 couples (46 in the study group and 52 in the control group) diagnosed with unexplained infertility who underwent IUI at Zeynep Kamil Women’s and Children’s Diseases Training and Research Hospital between 2021 and 2022. The study protocol was approved by the Clinical Research Ethics Committee of the institution with reference number EY.FR.22/111, dated August 23, 2023. After obtaining ethical approval, a total of 122 cycles (53 in the study group and 69 in the control group) were evaluated for chemical pregnancy, clinical pregnancy, and live birth outcomes. The early follicular phase levels of follicle-stimulating hormone (FSH) (mIU/mL), luteinizing hormone (LH) (mIU/mL), estradiol (pg/mL), thyroid stimulating hormone (TSH) (µIU/mL), and prolactin (ng/mL) were compared between the groups.

Semen analysis was performed according to the World Health Organization (WHO) criteria, with sperm concentration (million/mL), volume (mL), and progressive motility percentage (%) being assessed [7]. The study was divided into two groups based on the TPMSC of male partners: below 5 million and 5 million or above. The impact of TPMSC on pregnancy outcomes was evaluated. The couples who underwent controlled ovarian hyperstimulation resulting in the development of a single follicle were included in both the study and control groups for IUI treatment.

The inclusion criteria included patients aged between 21 and 40 years, primary or secondary infertility diagnosis, TPMSC either above or below 5 million, FSH level below 10 mIU/mL, prolactin level below 50 ng/mL during the early follicular phase, no prior intracytoplasmic sperm injection (ICSI) treatment, absence of hydrosalpinx, and negative tests for anti-HCV, anti-HIV, and HBsAg.

The exclusion criteria included patients aged between 18 and 21 years, FSH level above 10 mIU/mL, male partners with azoospermia, prolactin levels above 50 ng/mL in the early follicular phase, positive tests for anti-HCV, anti-HIV, or HBsAg, and female partners with hydrosalpinx. The inclusion and exclusion criteria are summarized in Table 1.

**Table 1:** Inclusıon and exclusion criteria of the study TPMSC: total progressive motile sperm count, FSH: follicle-stimulating hormone, HCV: hepatitis C virus

Inclusion criteria	Exclusion criteria
Ages between 21 and 40 years	Patients aged between 18 and 21 years
Primary or secondary infertility diagnosis	FSH level above 10 mIU/mL
TPMSC above or below 5 million	Male partners with azoospermia
FSH level below 10 mIU/mL	Prolactin levels above 50 ng/mL in the early follicular phase
Prolactin level below 50 ng/mL during the early follicular phase	Positive tests for anti-HCV, anti-HIV, or HBsAg
No prior intracytoplasmic sperm injection (ICSI) treatment	Female partners with hydrosalpinx
Absence of hydrosalpinx	
Negative tests for anti-HCV, anti-HIV, and HBsAg	

For ovulation induction, recombinant FSH or urinary FSH was administered, starting at a dose of 75 IU. Patients were monitored with transvaginal 2D ultrasound every two to three days to observe follicular growth, and gonadotropin doses were increased by 37.5 IU as necessary. After a dominant follicle was obtained, ovulation was triggered with hCG. If estradiol levels exceeded 2000 pg/mL or more than three follicles were >14 mm, the cycle was canceled to reduce the risk of multiple pregnancies and ovarian hyperstimulation syndrome (OHSS). IUI was performed 36 hours after the ovulation trigger. After IUI, patients rested in a supine position for 15 minutes, and prophylactic vaginal progesterone (200 mcg) was administered. A beta human chorionic gonadotropin (hCG) test was performed 14 days after IUI to diagnose chemical pregnancy, with a value above 1.2 mIU/mL indicating pregnancy. Clinical pregnancy was diagnosed when a gestational sac and fetal heartbeat were visible on 2D ultrasound, and live births were considered those pregnancies that reached 24 weeks and resulted in a live birth.

TPMSC was calculated by dividing sperm concentration (million/mL) by 100 and multiplying by semen volume (mL) and the percentage of motile sperm (%). Cases with TPMSC below 5 million were classified as severe oligoasthenospermia, and it was considered that significant IUI success could not be achieved in these cases.

Statistical analysis

Descriptive statistics for continuous variables included mean, standard deviation, median, minimum, and maximum values. Frequencies (n) and percentages (%) were calculated for categorical variables. The normality of the data was assessed using the Kolmogorov-Smirnov test. For non-normally distributed continuous variables, the Mann-Whitney U test was used to compare the two groups. When normality assumptions were met, an independent samples t-test was applied. Relationships between categorical variables were examined using the Chi-square or Fisher's exact test. All analyses were performed using IBM SPSS version 25 (IBM Corp., Armonk, NY) and a significance level of p < 0.05 was considered statistically significant.

## Results

This retrospective study included 122 IUI cycles from 98 couples diagnosed with unexplained infertility who underwent IUI at the Zeynep Kamil Women’s and Children’s Diseases Training and Research Hospital Infertility Clinic between 2021 and 2022. Among these cycles, 69 were from male partners with a TPMSC of 5 million or more, while 53 were from male partners with a TPMSC of less than 5 million.

The demographic and laboratory data for the patients who underwent IUI per cycle, including mean, standard deviation, median, minimum, and maximum values, are shown in Table 2. The mean age of the 98 female partners was 28.79 ± 4.33 years, with a mean age of 31.83 ± 4.37 years for the male partners. The average basal FSH level was 6.00 ± 1.49 mIU/mL, and the average basal LH level was 6.27 ± 3.52 mIU/mL. The mean values for basal estradiol (E2), 38.80 ± 14.18 pg/mL; prolactin (PRL), 20.19 ± 7.63 ng/mL; and TSH, 2.21 ± 1.07 µIU/mL were also recorded.

**Table 2:** Demographic characteristics and laboratory data of patients who underwent IUI in the hospital (n=98) FSH: follicle-stimulating hormone, LH: luteinizing hormone, E2: estradiol, PRL: prolactin, TSH: thyroid-stimulating hormone, IUI: intrauterine insemination

Parameters	Mean	Median (range)
Female age	28.79 ± 4.33	27.50 (22.00 - 40.00)
Male age	31.83 ± 4.37	31.00 (25.00 - 40.00)
Basal FSH (mIU/mL)	6.00 ± 1.49	5.88 (2.65 - 9.50)
Basal LH (mIU/mL)	6.27 ± 3.52	5.48 (1.23 - 25.50)
Basal E2 (pg/mL)	38.80 ± 14.18	35.95 (15.20 - 80.00)
Basal PRL (ng/ml)	20.19 ± 7.63	19.70 (7.20 - 46.50)
Bazal TSH (mU/L)	2.21 ± 1.07	2.13 (.30 - 5.86)

The mean sperm concentration was 28.05 ± 29.36 million/mL, sperm volume was 2.33 ± 1.32 mL, the percentage of progressively motile sperm was 46.93 ± 24.14%, and the average TPMSC was 31.73 ± 34.3 million. The average number of induced days was 10.10 ± 3.70 days, and the average total gonadotropin dose was 787.93 ± 353.63 IU. The average number of attempts was 1.22 ± 0.49, the average infertility duration was 3.59 ± 2.86 years, and the mean starting dose was 74.69 ± 8.11 IU.

In the group with TPMSC below 5 million, the mean age of female patients (29.83 ± 4.63 years) was significantly higher than that of the group with TPMSC above 5 million (27.87 ± 3.87 years) (p = 0.048, t = -2.00, df = 95). The mean basal FSH of the TPMSC below 5 million group (6.59 ± 1.55 mIU/mL) was significantly higher than that of the TPMSC above 5 million group (5.48 ± 1.24 mIU/mL) (p < 0.001, t = -4.09, df = 95, Cohen’s d = 0.84). The mean basal prolactin (PRL) in the TPMSC below 5 million group (22.07 ± 6.92 ng/mL) was significantly higher than that in the TPMSC above 5 million group (18.53 ± 7.91 ng/mL) (p = 0.004, t = -2.88, df = 95, Cohen’s d = 0.59).

Comparing the sperm parameters between the two groups, the sperm concentration (million/mL) in the TPMSC below 5 million group (7.23 ± 8.17 million/mL) was significantly lower than in the TPMSC above 5 million group (44.04 ± 29.76 million/mL) (p < 0.001, t = -7.68, df = 95, Cohen’s d = 1.56). Additionally, the number of induced days in the TPMSC below 5 million group (8.83 ± 2.56 days) was significantly lower than in the TPMSC above 5 million group (11.07 ± 4.15 days) (p = 0.003, t = -2.98, df = 95, Cohen’s d = 0.61). Similarly, the total gonadotropin dose in the TPMSC below 5 million group (649.49 ± 201.70 IU) was significantly lower than in the TPMSC above 5 million group (894.28 ± 406.20 IU) (p = 0.001, t = -3.45, df = 95, Cohen’s d = 0.71).

Despite these findings, there was no significant difference between the groups regarding the number of attempts, infertility duration, or starting dose (p > 0.05).

The chemical pregnancy rate was significantly higher in the group with TPMSC above 5 million (34.8%) compared to the group with TPMSC below 5 million (0.0%) (p < 0.001, χ² = 47.52, df = 1, Cramér’s V = 0.63), as shown in Table 3. Similarly, the clinical pregnancy rate was significantly higher in the TPMSC above 5 million group (31.9%) than in the TPMSC below 5 million group (0.0%) (p < 0.001, χ² = 44.50, df = 1, Cramér’s V = 0.61). The live birth rate was also significantly higher in the TPMSC above 5 million group (23.2%) compared to the TPMSC below 5 million group (0.0%) (p < 0.001, χ² = 38.62, df = 1, Cramér’s V = 0.57). No significant difference was found in the ages of the patients and their partners between the pregnant and non-pregnant groups (p > 0.05). Additionally, there were no significant differences between the pregnant and non-pregnant groups in terms of total induced days, total gonadotropin dose, and the number of attempts (p > 0.05).

**Table 3:** Comparison of pregnancy rates according to total progressive motile sperm count TPMSC: total progressive motile sperm count

	TPMSC 5 million < (n=53)	TPMSC 5 million > (n=69)	p
Biochemical pregnancy	0	24	<0.001
Clinical pregnancy	0	22	<0.001
Live birth	0	16	<0.001

The sperm concentration (22.13 ± 24.17 million/mL) in the non-pregnant group was significantly lower than in the pregnant group (52.22 ± 36.21 million/mL) (p < 0.001, t = -5.62, df = 95, Cohen’s d = 1.16). Similarly, the percentage of progressively motile sperm (43.20 ± 24.25%) in the pregnant group was significantly lower than in the non-pregnant group (62.17 ± 16.90%) (p < 0.001, t = -6.34, df = 95, Cohen’s d = 1.29). However, there was no significant difference in sperm volume between the pregnant and non-pregnant groups (p > 0.05).

In the group with TPMSC above 5 million, there was a significant relationship between infertility type and pregnancy outcome. The chemical pregnancy rate in the secondary infertility group (62.5%) was significantly higher than in the primary infertility group (26.4%) (p = 0.008, χ² = 7.12, df = 1, Cramér’s V = 0.26), as shown in Table 4.

**Table 4:** Comparison of infertility types and pregnancy rates according to ≥ 5 million TPMSC TPMSC: total progressive motile sperm count

Infertility type	p, No(n=45)	p, Yes (n=24)	Total (n=69)	p
Primary	39 (73.6%)	14 (26.4%)	53	0.008
Secondary	6 (37.5%)	10 (62.5%)	16	

No significant differences were observed between the pregnant and non-pregnant groups in terms of sperm concentration (million/mL), sperm volume (mL), and the percentage of progressively motile sperm within the TPMSC above 5 million group (p > 0.05). However, sperm concentration was approximately twice as high in the pregnant group compared to the non-pregnant group.

Lastly, when patients in the TPMSC above 5 million group were further categorized, no significant relationship was found between TPMSC and chemical pregnancy, clinical pregnancy, and live birth rates (p > 0.05), as shown in Table 5.

**Table 5:** Comparison of pregnancy rates according to the TPMSC above 5 million TPMSC: total progressive motile sperm count

	TPMSC 5-19.99 million	TPMSC ≥ 20 million	p
Biochemical pregnancy	3 (33.3%)	21 (35%)	1
Clinical pregnancy	3 (33.3%)	19 (31.7%)	1
Live birth	3 (33.3%)	13 (21.7%)	.423

## Discussion

IUI is widely used with or without ovulation induction, but its effectiveness varies. Guzick et al. reported a pregnancy rate of 17.1% per cycle with gonadotropin plus IUI, while Goverde et al. and the European Society of Human Reproduction and Embryology (ESHRE) subgroup reported 8.7% and 12%, respectively [8-10]. Our study found a pregnancy rate of 19.7%, which aligns closely with the results of Guzick et al. [8]. Age has been consistently shown to affect IUI success. Stone et al. identified age as a key predictor, noting a decline in success after age 32 [11]. Our study found no significant difference in pregnancy rates related to age (29.29 ± 4.12 years in the pregnant group, p > 0.05), contrasting with the significant findings of Stone et al. [11]. This could be due to the younger average age in our cohort.

The relationship between TPMSC and IUI success has been well documented. Miller et al. highlighted that a TPMSC above 5 million significantly improves pregnancy chances. In our study, the group with TPMSC above 5 million had 24 pregnancies, while no pregnancies were observed in the group with TPMSC below 5 million (p < 0.01) [12]. These findings are consistent with Miller et al., who found lower success in couples with TPMSC under 10 million [12]. Our research further supports the conclusion that TPMSC is a critical factor in predicting pregnancy outcomes in IUI cycles.

Semen volume, sperm concentration, and motility are also crucial sperm parameters for predicting pregnancy success. While no significant difference was found in semen volume between groups (p > 0.05), sperm concentration and motility were significantly higher in the pregnant group (p < 0.01), which mirrors findings by Miller et al. [12]. Ombelet et al. reported that TPMSC thresholds below 5 million are more predictive of IUI success than values between 5 and 20 million, a finding supported by our study [13].

Secondary infertility is another factor that can influence IUI outcomes. Dorjpurev et al. and Kamath et al. did not find significant differences in pregnancy rates between primary and secondary infertility couples [14,15]. However, in our study, pregnancy rates were higher among secondary infertility couples (62.5%) compared to primary infertility couples (26.4%) when TPMSC was above 5 million, although this difference was not statistically significant (p = 0.08). This result is consistent with studies by Dickey et al., who also found higher success rates in secondary infertility but with14 varying statistical significance [16].

Dickey et al. established that progressive motility and total motile sperm count were strongly correlated with IUI success [16]. They found that a sperm concentration of ≥ 5 x 10^6/ml, total sperm count of ≥ 10 x 10^6/ml, progressive motility ≥ 30%, or total motile sperm count ≥ 5 x 10^6 were associated with pregnancy rates ≥ 8.2%. Although the group with TPMSC below 5 million had sperm concentration and motility values exceeding the thresholds established by Dickey et al., no pregnancies occurred in this group, underscoring the significance of using a 5 million TPMSC cutoff when predicting IUI success [16].

In conclusion, our findings, in line with the studies of Miller et al. and Dickey et al., emphasize that TPMSC is a critical predictor of IUI success [12,16]. While a previous study suggested a 10 million TPMSC threshold, our study demonstrates that 5 million is a more effective cutoff for predicting successful IUI outcomes [12].

Study limitations

This study has several limitations. It is a retrospective analysis, and data were collected from existing records. The group with TPMSC below 5 million (53 cycles) had a smaller sample size, limiting the generalizability of the findings. Conducted at a single center, the results may not be applicable to different populations or settings, and future multi-center studies are needed. The study focused only on couples with unexplained infertility, so the effects of TPMSC in other infertility types remain unclear. Additionally, the study assessed only pregnancy outcomes and did not follow long-term health outcomes of live births. Other factors influencing sperm quality, such as hormonal profiles, lifestyle, and genetics, were not considered. Future research should include larger, prospective studies with long-term follow-up.

## Conclusions

In this study, we explored the role of TPMSC in predicting pregnancy outcomes for couples with unexplained infertility undergoing IUI. Our findings indicate that a TPMSC of 5 million or more significantly improves the chances of pregnancy. This emphasizes the importance of defining an optimal TPMSC threshold for predicting IUI success, as patients with severe oligoasthenospermia may benefit more from transitioning directly to more advanced assisted reproductive technologies, such as IVF, to improve their chances of conception.

Our results support the notion that focusing treatment on patients with a TPMSC above 5 million may lead to better clinical outcomes while avoiding the emotional, physical, and financial toll of multiple unsuccessful IUI cycles for couples with lower sperm motility. This study also highlights the need for a more cost-effective and targeted approach in treating couples with unexplained infertility, particularly in cases where IUI is unlikely to succeed. Ultimately, further studies are necessary to refine TPMSC thresholds and optimize treatment protocols for unexplained infertility patients.
